# HM6: greater than the sum of its parts?

**DOI:** 10.1093/icvts/ivad139

**Published:** 2023-08-31

**Authors:** Mackenzie Adcox, Ioannis Dimarakis

**Affiliations:** Division of Cardiothoracic Surgery, Department of Surgery, University of Washington Medical Center, Seattle, WA, USA; Division of Cardiothoracic Surgery, Department of Surgery, University of Washington Medical Center, Seattle, WA, USA

**Keywords:** RVAD, BiVAD, Methods

The prevalence of right ventricular failure (RVF) after left ventricular assist device (LVAD) implantation has been estimated at ∼35% [1]. RVF post-LVAD implantation has long been shown to confer significant morbidity and mortality, leading to increased ICU length of stay and lower bridge-to-transplant rate [2]. Despite appropriate medical management, it is estimated that ∼10–15% of patients with RVF post-LVAD implantation will require some form of mechanical RV support [3]. Temporary RV mechanical support (t-RVAD) can be instituted surgically or percutaneously. In a recent systematic review, Abdelshafy *et al.* [4] showed that t-RVAD wean was quite variable among studies at 23–100% with a reported switch to permanent RVAD support ranging between 7% and 35%. Although data for optimal timing for t-RVAD implantation are limited, the majority of studies suggest that concurrent (with LVAD) versus delayed t-RVAD support may confer a survival benefit [5, 6].

Regardless of the timing of t-RVAD placement, there is undeniably an especially challenging subset of patients in whom wean from t-RVAD support is not tolerated and will require ongoing, durable right ventricular support. The total artificial heart (TAH) option has many limitations based on its large size, large pneumatic drive system limiting mobility, and geographic availability [7]. An alternative surgical strategy is the use of a second durable VAD support device in a configuration to support the right ventricle. In comparison to TAH, the advantage of the ‘HeartMate 6’ approach is that HM3 (Abbott, Abbott Park, IL) devices are widely available, relatively small and noiseless. At the single institutional level, HM3 BiVAD has been reported with low mortality [8], with a similar bridge-to-transplant outcomes compared to TAH [9].

Milano *et al.* [10] reported in 2015 that over 100 patients had implanted 2 HVAD pump (Heart-Ware Inc, Framingham, MA) pumps placed for biventricular support. Thus far, 2 distinct approaches to durable ‘BiVAD’ implantation have been described: RVAD sewing ring attached to the right atrium or right ventricle with standard LVAD configuration or alternatively, complete biventriculectomy with sewing rings attached to the tricuspid and mitral valve apparatus, respectively [10]. Urganci *et al.* [11] have shared their approach of the ‘HeartMate 6’ where 2 HM3 devices were implanted in a virgin chest of a patient with Yamaguchi syndrome. Their technique involved complete biventriculectomies and excision of atrioventricular valves. In turn, the sewing rings were sutured to each atrium and respective outflow grafts were anastomosed to the pulmonary artery and aorta sequentially. Daneshmand *et al.* [7] describe a similar operative technique with biventriculectomies in a patient with RVF following LVAD reconfiguration due to persistent driveline infection.

Herein, the Berlin group describes a patient who presents with biventricular failure due to ischaemic cardiomyopathy and ultimately is taken for HM3 LVAD implantation [12]. The decision was made intra-operatively to implant a CentriMag (Levitronix LLC, Waltham, MA) t-RVAD. The initial postoperative course was unremarkable; however, the patient was unable to wean from t-RVAD support. As such, the team moves forward with implanting a second HM3 device in the RVAD configuration. In the video, one can appreciate the extensive adhesions depicting the significant challenge seen with re-do surgery in this early timeframe. As a result of the hostile mediastinum, the team utilizes 3 critical techniques: femoral cannulation for cardiopulmonary bypass, placement of the fixation ring on the pericardium itself and sewing the outflow graft in an end–end anastomosis with the distal end of the *in situ* t-RVAD graft. In addition, the team eloquently show a technique of ring augmentation to make up the discrepancy between inflow cannula length and right atrial size.

Authors make use of excellent visuals to contextualize the intraoperative challenges in this complex clinical scenario and provide simple techniques used to mitigate them. Clearly not for the ‘faint-hearted’, this approach is becoming an important addition to the armamentarium of every practicing mechanical circulatory support surgeon.
